# Hay Fever: Its Cause and Cure

**Published:** 1885-07

**Authors:** E. Fletcher Ingals

**Affiliations:** Professor of Laryngology, Rush Medical College; Professor of Diseases of the Throat and Chest, Woman’s Medical College, Chicago; 64 State Street, Chicago


THE CHICAGO
MEDICAL JOURNAL AND EXAMINER.

VOL. LI. JULY, 1885. No. 1

### ORIGINAL COMMUNICATIONS.

---

HAY-FEVER: ITS CAUSE AND CURE. *By* E. FLETCHER INGALS, A. M., M. D., *Professor of Laryngology, Rush Medical College; Professor of Diseases of the Throat and Chest, Woman's Medical College, Chicago.*

[Read before the Chicago Medical Society, 25th May.]

Synonyms—Hay-fever, Hay-asthma, Rose-cold, June-cold, Idiosyncratic Coryza, Autumnal Catarrh, etc.

The subject of this paper is one which has been before the profession since it was discovered by Bostock in 1819, and within the last twenty-five years it has been the subject of careful research by numerous diligent workers; however, it is only recently that anything like a satisfactory solution of the difficulties attending its treatment have been reached.

The disease is met with commonly in the southern part of England, but is comparatively rare in other parts of Europe, while in Asia and Africa it is said that only Europeans are affected by it. In the United States it is a very common affection which drives thousands from their homes every year, and makes miserable many more who are unable to make the change of climate which has hitherto been the only remedy for the malady.

Notwithstanding the generally received opinion that it is caused by the pollen of certain grains, examples of it are much less frequent among those living upon farms, where they are constantly exposed to the pollen, than among those living in villages, and these latter are less likely to suffer from it than the residents of cities.

This might, with considerable propriety, be called an aristocratic affection, as it seldom attacks any but those in comparatively easy circumstances, though it occasionally occurs among others. I have seen a few well-marked examples among the laboring class.

In this country, hay-fever usually makes its appearance about the middle of August and continues until the first frosts of the autumn, but in England it is most prevalent in June and July. However, numerous cases are observed in the early spring, and isolated cases, which might be better defined by the term, idiosyncratic catarrh, are occasionally seen in the winter months. These latter, though not excited by pollen are clearly due to similar conditions in the patient, which favor the development of attacks from a considerable variety of exciting causes.

During an attack of hay-fever, the mucous membrane covering the turbinated bodies is greatly swollen and usually, though not always, congested, and the congestion often extends to the palate, fauces, pharynx and conjunctivæ. Microscopic examinations of the secretions have discovered numerous vibriones, which by many, following the theory of Helmholtz*,

* Virchow's Archives. Feb., 1869.

have been thought to be the cause of the affection; but as the same objects may usually be found in the secretions of the nasal cavities at other times, it is thought that the presence of these is merely accidental. Various substances floating in the atmosphere have been found to act upon the respiratory mucous membranes of certain individuals in such manner as to give rise to the train of symptoms, which taken together represent the affection known as hay-fever. These substances do not cause the affection *per se*, but merely act as irritants, for any of them indiscriminately may affect some individuals, while only certain ones can excite the affection in others. The substances which have been found to be the most common exciting causes of the affection are the pollen of the grammaceæ which is found abundantly in the air in England during the months of June and July, when the disease is most prevalent, and in America by that of Ambrosia Artemisiæfolia, (ragweed or hogweed) when the plant is in full bloom during the months of August and September.

But besides these, the pollen of rye, wheat, oats, Indian corn and other grains are active with some persons, and still other agents may produce similar effects in patients affected by peculiar idiosyncrasies. For example, dust and the emanations from various drugs, flowers, animals, etc. These latter circumstances explain the origin of the peculiarly appropriate term idiosyncratic coryza.

These and other facts in the clinical history seem strongly to support the theory that cases of so-called catarrh in which the slightest provocation, such for example, as passing from a warm to a cold room or the reverse, or passing from the shade into the bright sunlight will excite frequent sneezing, as neither more nor less than examples of imperfectly developed idiosyncratic coryza or, as commonly known, hay-fever.

Why the substances mentioned should affect certain individuals and not others is a question which has engaged the attention of the profession for a long time, but it has only recently been satisfactorily answered. The more or less severe attacks in the same individual which may at different times be brought on by the same substance are analogous to the varying severity of the attacks of neuralgia which may be excited by exposure to cold; and, as in the latter instance, they undoubtedly result from the condition of the nervous system instead of from any special quality in the exciting agent.

When the nervous system is exhausted by over work, loss of rest, or imperfect nutrition, it becomes irritable and then agencies, which during perfect health might be innocuous, will in one excite neuralgia; in another an acute catarrh, and in a third a well developed attack of hay-fever,—the peculiar manifestation being determined by a peculiar susceptibility of certain nerves, which from some unknown changes, become acutely sensitive.

The theory of a nervous origin of hay-fever has been generally accepted by the profession, but it was vaguely referred to the whole nervous system and until recently no one suspected what now seems an established fact, viz., that the tract involved is sharply defined and usually more limited than that in a typical case of intercostal neuralgia.

In 1882, Daly*, of Pittsburgh, first called attention to the peculiar sensitiveness of certain portions of the nasal mucous membrane in individuals suffering from hay-fever, and later, clinical observations of Roe,[1] of Rochester, N. Y.; Sajous,[2] of

---

*Archives of Laryngology, Vol. II.

1. N. Y. Medical Journal, May, 1883.

2. Medical and Surg. Reporter, December, 1883.

Philadelphia; Hack,[1] of Freiborg; and Allen,[2] of Philadelphia, and the physiological experiments of Mackenzie,[3] of Baltimore, localized the sensitive tract for the majority of cases on the inferior turbinated bodies and the lower and back part of the septum. In a few individuals much more of the nasal mucous membrane is involved, and it is probable that, in very rare cases, the sensitive tract will be found to extend to the fauces and possibly to the bronchial mucous membrane, though usually during an attack the involvement of the pharyngeal and bronchial mucous membranes is due to the extension of the inflammation from the part first affected.

The later experience of the physicians just mentioned and that of several others, with my own observations, leave no doubt in my mind that the theory rests on a foundation of fact. In the majority of cases the posterior third of the inferior turbinated bodies and the lower part of the posterior two-thirds of the septum are the only sensitive parts, but in a considerable number of cases the sensitive parts are even more limited, though in some the middle turbinated body and the anterior portions of the septum and lower turbinated bodies are also involved.

The sensitive areas are not uniform in different cases. They are commonly found in patches which may be either in one or both nares, and, in some, they may be found on any of the parts already described.

Some years ago, Professor Helmholz found certain vibriones in the secretions from the nares of hay-fever patients which he supposed were the cause of the disease; and he concluded that the local application of quinine, or other agents destructive

---

1. Wein. Med. Worchen., August, 1883.
2. Am. Jour. of Med. Science, January, 1884.
3. N. Y. Med. Record, July, 1884.

to these minute organisms, ought to cure the affection. This treatment has been extensively employed and numerous cases have been relieved; but unfortunately for the theory it has been shown that not only weak solutions of quinine but also those of soda or other non-irritating substances are equally efficacious, the germicidal properties of the solution having nothing to do with the result. The relief therefore must be attributed to cleansing of the nares from the pollen or other irritating particles or to the soothing influence of the remedy upon the terminal nerve-fibers in the mucous membrane.

Clinical experience points to the nervous origin of the affection and locates the principal changes in the branches of the spheno-palatine ganglion and nasal nerves; but we are unable to discover in what these changes consist. They seem, however, analagous to the condition in neuralgia and hyperæsthesia.

As a result of irritants coming in contact with these sensitive surfaces some change takes place in the vaso-motor nerve which is shown by sudden swelling of the erectile tissues in the nares. But substances causing hay-fever seem to act in some way differently from ordinary irritants, for, notwithstanding the swelling, in some cases there is no congestion whatever of the mucous membrane.

The primary effect of the irritation is to cause profuse secretion from the nares with swelling of the mucous membrane. This partially or wholly occludes the nares and necessitates mouth-breathing, with consequent irritation of the throat. The asthmatic symptoms which attend a considerable number of cases may be caused directly by irritation of the bronchial mucous membrane, but more probably they are usually the reflex result of the nasal obstruction and irritation.

Prolonged contact of the irritant will, in most cases, set up an acute inflammation of the Schneiderian mucous membrane, which may extend to the mucous membrane of the eyes, ears, throat and bronchi.

In this country patients are usually attacked about the middle of August, and they soon learn to foretell the time of the attack almost to the hour. Generally the first symptoms are similar to those of a cold in the head, attended by frequent sneezing and burning pain with profuse secretion from the nose and eyes. Rarely the first symptoms are referable to the naso-pharynx and fauces. The eyes and nose soon become red, swollen and tender, and usually the temperature is slightly elevated. There is sometimes extreme suffering. Very soon nasal respiration is greatly interfered with, and in many cases asthmatic attacks add to the discomfort.

During the height of the attack the profuse discharge frequently causes erosion of the nostrils and upper lip; the conjunctivæ are occasionally much inflamed and the fauces are more or less congested. At the same time examination of the chest will often discover numerous bronchial rales. These symptoms ordinarily continue with greater or less severity from two to six weeks. In some cases they do not disappear until the first sharp frosts of the ensuing autumn.

In those who have previously suffered from the disease there is little difficulty in making a correct diagnosis; but in first attacks the affection may be readily confounded with an ordinary severe cold in the head. The principal points to notice in forming a diagnosis are: the periodicity of the disease, its sudden accession, the sensitiveness and swelling of the nasal mucous membranes; the asthmatic symptoms and the obstinacy of the attack as compared with an ordinary cold or spasmodic asthma.

The affection is not of itself serious though from suffering and loss of rest the patient may become so exhausted as to fall an easy prey to other disease. Each attack may be expected to continue for several weeks only to disappear with the change in the season ; and the disease may be confidently expected to recur for many years ; though with some the tendency to it gradually disappears.

In the treatment of this affection as in that of all other intractable, self-limited diseases, many remedies have from time to time been vaunted as specifics, but all medicinal measures have been found sadly wanting in curative properties. Many times remedies have been employed in individual cases with apparently good results ; but upon the next case in which they have been tried they have either proven inert or actually injurious. This is readily understood if we consider that without any treatment the symptoms sometimes speedily disappear. In the first case the remedy may have been given just as convalescence was to begin ; while in the second the person may have been growing worse at the time.

Of a vast number of medicines recommended for internal or local use I have not time to speak ; but I may safely say that with few exceptions, which I will soon mention, they are of very little value and we should not encourage patients to expect relief from them ; though they may be tried in cases where the proper treatment cannot be carried out. Of the drugs which may be employed internally with a hope of warding off or lessening the severity of an attack, quinine, strychnine, arsenic and iron are of more or less value, from their influence in promoting the general health and building up the nervous system. Valerian, assafœtida and phosphide of zinc are also useful, for their anti-spasmodic effects.

In the inception of the attack benefit has been derived from the local use of a weak solution of quinine, carbolic acid or tincture of opium, but these remedies will only help a small number of cases and they may utterly fail, even in these, on a second trial. These same remedies are recommended for the fully developed disease but usually they give very little relief.

Sedative powders composed of morphia, bismuth, iodoform, starch, etc., have sometimes given relief, while in other instances they have greatly aggravated the patient's sufferings.

For the asthmatic symptoms we may employ the same measures, which have been found useful in simple spasmodic asthma, chief among which are the inhalation of the fumes of nitre-paper, stramonium, hyoscyamus, etc., and the internal use of morphia and chloral ; but they will be found much less useful than for the simple spasmodic affection. In a word it may be said that the medicinal treatment of hay-asthma is in the highest degree unsatisfactory. As a result, all patients who can, resort to climatic treatment. In a large portion of cases this is effective in preventing the attack or at least in modifying its severity. However, the inconvenience of this course and the impossibility in many cases of having it followed have made physicians untiring in their efforts to discover some other means of preventing or curing the disease. These efforts have been rewarded by the discovery of a method of treatment which we believe will cure the disease in nine cases out of ten, if carried out before the attack begins. We have also found a remedy which promises much for the relief of fully developed cases.

A few weeks after the discovery of the anæsthetic properties of cocaine laryngologists* called attention to the peculiar

*Dr. Bosworth, of N. Y., was the first to point out this peculiarity, which he did in the N. Y. Medical Record, Nov. 15th, 1884; but before his article appeared I had already sent to press an account of my first experiments with the new anaesthetic, which was published in the Journal of the American Medical Association of the following week. In both these articles this property of the drug referred to and its application suggested in hay-fever and other affections which cause swelling of the nasal mucous membrane.

property possessed by the drug of causing speedy contraction of swollen mucuous membrane in the nares when applied in solution, or in the form of a powder. At the same time it was suggested that cocaine would probably relieve many of the distressing symptoms of hay-fever, but as yet no typical case has occured on which it might be tried. However, it has been used with great success to relieve the annoyance caused by swelling of the turbinated bodies in acute and chronic rhinitis, and from a large experience with it in these affections, I feel, confident, that it will be of value in the treatment of the disease now under consideration.

Although typical hay-fever only occurs in the summer or fall months, isolated cases of idiosyncratic coryza are met with in early spring, or even in winter; which from their peculiar course and close resemblance to typical cases of hay-fever, seem to be identical with the latter excepting in the accidental exciting cause. These cases are attented by severe paroxysms of sneezing, with coryza and lachrymation, stuffing up of the nose and sometimes paroxysms of asthma—symptoms exactly like those of ordinary cases of hay-fever.

One such case came under my observation during the past winter, which although having no asthmatic breathing was in every other respect similar to hay-fever. In this case I gave the patient a 2% mixture of cocaine with starch, which was blown into the nose as soon as the paroxysm came on. It gave immediate and perfect relief, though it did not at once cure the attack, which lasted for several days. In this patient the paroxysms came on in the evening, but a single application was usually sufficient to check it at once; however, sometimes it was necessary to repeat the application three or four times during the night, the symptoms being relieved each time within half a minute. Another attack came on a couple of weeks later which was promptly relieved in the same manner. This is the only case of idiosyncratic coryza that I know of which has been treated by this remedy; however, when considered with a large number of cases of hypertrophic catarrh, which I have relieved in the same manner, it gives me great confidence in recommending cocaine, for its temporary effects in hay-fever.

In hypertrophic catarrh I have found that patients who are obliged to use the remedy frequently, will, after a few weeks, cease to derive much benefit from it; though if discontinued for a time it will again act well. I presume the action would be similar in hay-fever patients. I conclude that although cocaine is a palliative agent of wonderful power, we cannot expect it to cure the disease.

Successful treatment by the galvano-cantery, which was first thorougly tried by Dr. Roe, had, previous to last May, been applied by Drs. Roe, Allen and Sajous to about thirty cases, about 80 per cent. of which were reported cured, and the operators believed that all could have been cured if the treatment had been completed. During the past year other laryngologists have adopted the same treatment but its results have not been recorded, as most of the patients have not gone through a hay-fever season since the treatment. Many of the patients who were treated last summer began the treatment so late that it could not be completed before the attack came on and therefore they did not escape. My own patients, whom I considered but partially cured, as a rule went through the season with much less discomfort than formerly, and the only one in whom I had time to carry the treatment to the end had no return of the disease.

Since last August I have treated several cases thoroughly, and judging from their present condition and the experience of others I expect them to go through the coming season without a recurrence of the attack.

As the disease is caused by a peculiar sensitiveness of the terminal branches of the spheno-palatine ganglion and nasal nerve which are distributed over the septum and turbinated bodies, the treatment to be effective must remedy this hyperæsthetic condition. The means adopted for accomplishing this consist of applications of glacial acetic acid, carbolic acid and other escharotics, and searing the membrane with the galvano-cantery.

Sometimes mucous polypi exist which must be removed; hypertrophy of the turbinated bodies may be present, which must be connected or the spur of a deflected and thickened septum must be sawed off. The treatment by chemical agents will sometimes succeed but will often fail, while the galvano-cantery properly used cannot fail to remove the excessive sensibility and leave the membrane in a healthy condition. Successful treatment usually requires from ten to twenty sittings and should be completed before the attack comes on, therefore, as an interval of three to five days is desirable between each sitting, the treatment should be begun early in the season.

The plan which I adopt, and which has been found so successful by others, is to sear slightly with the galvano-cantery every portion of the peculiarly sensitive membrane; using a small electrode and cauterizing but a small place at each sitting. I first carefully examine the nasal cavity with a slender, flat probe by which the sensitive spots are located. I then pass the cold electrode into the naris, and having reached the spot to be cauterized, turn on the electricity, whereby the wire is instantly heated. It is then applied for a fraction of a second to the diseased tissue, causing a small superficial burn. This operation is repeated at each subsequent sitting until all the sensitive spots have been relieved; care being taken not to burn too much at once lest it cause an uncomfortable inflammation. Even without an anæsthetic if a good battery* is used and proper electrodes, the pain caused by this operation is slight, in most cases, but some persons feel it severely for an instant. Heretofore, a few of these patients preferred to have the disease rather than suffer the treatment, and consequently they could not be cured; but fortunately the anæsthetic properties of cocaine now enable us to treat all cases without pain.

In applying the cocaine I use a 4 per cent. solution in distilled water, which I drop upon the surface from a small syringe at regular intervals of about two minutes. Usually the parts will become anæsthetized in twelve minutes, occasionally eight minutes is sufficient to produce anæsthesia, but frequently it will require twenty or thirty minutes and, rarely longer. In one case in which I was producing anæsthesia for a different purpose it required over an hour's time and about five grains of the cocaine.

After the cauterization, when no anæsthetic has been used a spray of Dobells' solution should be employed to relieve smarting. During the intervals between treatment patients should keep the nares cleaned by insufflations or spray of soda and water or Listerine. The latter is sometimes highly useful for its antiseptic properties.

In conclusion, the following points seem to me established with reference to the treatment of hay-fever.

1st. Nearly all cases may be cured by systematic, thorough, superficial cauterization of the hypersensitive portions of the nasal mucous membrane, providing the treatment is carried out during the interval between the attacks.

---

*I employ a powerful two-celled bichromate of potash battery which which was made for me by Sharp & Smith, of this city.

2nd. The most effective and least painful means of accomplishing this, is by the galvano-cantery.

3rd. Care must be exercised to treat every sensitive spot, and not to cauterize too large a surface at once.

4th. The operation may be made painless by a proper use of hydrochlorate of cocaine.

5th. In nervous subjects general treatment must not be neglected.

6th. The effects of cocaine in hypertrophic catarrh, and in the case of idiosyncratic coryza just reported, render it highly probable that it will give much relief in many cases of hay-fever.

64 State Street, Chicago.

